# Supplementary material for: Detection of EGFR mutations in non-small cell lung cancer by droplet digital PCR
Source: PLoS One. 2022 Feb 24;17(2):e0264201. doi: 10.1371/journal.pone.0264201 (PMC8870499; doi:10.1371/journal.pone.0264201)
Supplement: S1 Dataset — (DOCX) [file pone.0264201.s001.docx]

**ddPCR Experimental Data**

This set of experimental data was used to calculate analytical specificity, limit of detection (**Figure 4**), accuracy, and precision (**Figure 5**).

***EGFR* L858R assay**

DN - droplets double negative for VIC and FAM
WT - droplets positive for VIC only
MUT - droplets positive for FAM only
DP - droplets positive for VIC and FAM

| Well | VAF | Conc | DN | WT | MUT | DP | TOT | POS | P(MUT) | P(WT) | P(ALL) | λ(MUT) | λ(WT) | λ(ALL) | VAF(Pois) |
| --- | --- | --- | --- | --- | --- | --- | --- | --- | --- | --- | --- | --- | --- | --- | --- |
| A1 | 0% | 3.125 | 8186 | 1161 | 0 | 0 | 9347 | 0 | 0 | 0.124 | 0.124 | 0 | 0.133 | 0.133 | 0.0000% |
| A2 | 0% | 3.125 | 13160 | 1865 | 0 | 0 | 15025 | 0 | 0 | 0.124 | 0.124 | 0 | 0.133 | 0.133 | 0.0000% |
| B1 | 0% | 6.25 | 13754 | 4386 | 0 | 1 | 18141 | 1 | 5.51E-05 | 0.242 | 0.242 | 5.51E-05 | 0.277 | 0.277 | 0.0199% |
| B2 | 0% | 6.25 | 12794 | 3716 | 0 | 0 | 16510 | 0 | 0 | 0.225 | 0.225 | 0 | 0.255 | 0.255 | 0.0000% |
| C1 | 0% | 12.5 | 8848 | 6691 | 0 | 0 | 15539 | 0 | 0 | 0.431 | 0.431 | 0 | 0.563 | 0.563 | 0.0000% |
| C2 | 0% | 12.5 | 9714 | 6721 | 0 | 1 | 16436 | 1 | 6.08E-05 | 0.409 | 0.409 | 6.08E-05 | 0.526 | 0.526 | 0.0116% |
| D1 | 0% | 25 | 4955 | 9916 | 0 | 1 | 14872 | 1 | 6.72E-05 | 0.667 | 0.667 | 6.72E-05 | 1.099 | 1.099 | 0.0061% |
| D2 | 0% | 25 | 5836 | 10862 | 0 | 0 | 16698 | 0 | 0 | 0.65 | 0.65 | 0 | 1.051 | 1.051 | 0.0000% |
| E1 | 0% | 50 | 1527 | 12969 | 0 | 1 | 14497 | 1 | 6.9E-05 | 0.895 | 0.895 | 6.9E-05 | 2.251 | 2.251 | 0.0031% |
| E2 | 0% | 50 | 1346 | 6607 | 0 | 1 | 7954 | 1 | 0.000126 | 0.831 | 0.831 | 0.000126 | 1.777 | 1.777 | 0.0071% |
| F1 | 0.01% | 3.125 | 9752 | 1544 | 0 | 0 | 11296 | 0 | 0 | 0.137 | 0.137 | 0 | 0.147 | 0.147 | 0.0000% |
| F2 | 0.01% | 3.125 | 15073 | 2204 | 0 | 0 | 17277 | 0 | 0 | 0.128 | 0.128 | 0 | 0.136 | 0.136 | 0.0000% |
| G1 | 0.01% | 6.25 | 12032 | 3627 | 0 | 1 | 15660 | 1 | 6.39E-05 | 0.232 | 0.232 | 6.39E-05 | 0.264 | 0.264 | 0.0242% |
| G2 | 0.01% | 6.25 | 14471 | 4426 | 2 | 0 | 18899 | 2 | 0.000106 | 0.234 | 0.234 | 0.000106 | 0.267 | 0.267 | 0.0396% |
| H1 | 0.01% | 12.5 | 7972 | 7083 | 1 | 2 | 15058 | 3 | 0.000199 | 0.471 | 0.471 | 0.000199 | 0.636 | 0.636 | 0.0313% |
| H2 | 0.01% | 12.5 | 9032 | 7451 | 1 | 0 | 16484 | 1 | 6.07E-05 | 0.452 | 0.452 | 6.07E-05 | 0.602 | 0.602 | 0.0101% |
| A3 | 0.01% | 25 | 5002 | 11512 | 1 | 3 | 16518 | 4 | 0.000242 | 0.697 | 0.697 | 0.000242 | 1.194 | 1.195 | 0.0203% |
| A4 | 0.01% | 25 | 4705 | 10740 | 1 | 1 | 15447 | 2 | 0.000129 | 0.695 | 0.695 | 0.000129 | 1.189 | 1.189 | 0.0109% |
| B3 | 0.01% | 50 | 1377 | 12969 | 0 | 4 | 14350 | 4 | 0.000279 | 0.904 | 0.904 | 0.000279 | 2.344 | 2.344 | 0.0119% |
| B4 | 0.01% | 50 | 1412 | 15255 | 0 | 6 | 16673 | 6 | 0.00036 | 0.915 | 0.915 | 0.00036 | 2.469 | 2.469 | 0.0146% |
| C3 | 0.1% | 3.125 | 14915 | 2094 | 0 | 0 | 17009 | 0 | 0 | 0.123 | 0.123 | 0 | 0.131 | 0.131 | 0.0000% |
| C4 | 0.1% | 3.125 | 13615 | 2120 | 2 | 1 | 15738 | 3 | 0.000191 | 0.135 | 0.135 | 0.000191 | 0.145 | 0.145 | 0.1316% |
| D3 | 0.1% | 6.25 | 13385 | 4344 | 1 | 1 | 17731 | 2 | 0.000113 | 0.245 | 0.245 | 0.000113 | 0.281 | 0.281 | 0.0401% |
| D4 | 0.1% | 6.25 | 13446 | 4138 | 2 | 4 | 17590 | 6 | 0.000341 | 0.235 | 0.236 | 0.000341 | 0.269 | 0.269 | 0.1270% |
| E3 | 0.1% | 12.5 | 9811 | 7681 | 7 | 4 | 17503 | 11 | 0.000628 | 0.439 | 0.439 | 0.000629 | 0.578 | 0.579 | 0.1086% |
| E4 | 0.1% | 12.5 | 8850 | 6896 | 9 | 3 | 15758 | 12 | 0.000762 | 0.438 | 0.438 | 0.000762 | 0.576 | 0.577 | 0.1320% |
| F3 | 0.1% | 25 | 6122 | 11734 | 8 | 9 | 17873 | 17 | 0.000951 | 0.657 | 0.657 | 0.000952 | 1.07 | 1.071 | 0.0888% |
| F4 | 0.1% | 25 | 5101 | 11109 | 3 | 19 | 16232 | 22 | 0.001355 | 0.686 | 0.686 | 0.001356 | 1.157 | 1.158 | 0.1172% |
| G3 | 0.1% | 50 | 1808 | 14619 | 4 | 35 | 16466 | 39 | 0.002369 | 0.89 | 0.89 | 0.002371 | 2.207 | 2.209 | 0.1073% |
| G4 | 0.1% | 50 | 1355 | 14433 | 4 | 28 | 15820 | 32 | 0.002023 | 0.914 | 0.914 | 0.002025 | 2.455 | 2.457 | 0.0824% |
| H3 | 1% | 3.125 | 15466 | 2356 | 18 | 3 | 17843 | 21 | 0.001177 | 0.132 | 0.133 | 0.001178 | 0.142 | 0.143 | 0.8237% |
| H4 | 1% | 3.125 | 15765 | 2199 | 27 | 4 | 17995 | 31 | 0.001723 | 0.122 | 0.124 | 0.001724 | 0.131 | 0.132 | 1.3032% |
| A5 | 1% | 6.25 | 14199 | 4125 | 34 | 9 | 18367 | 43 | 0.002341 | 0.225 | 0.227 | 0.002344 | 0.255 | 0.257 | 0.9107% |
| A6 | 1% | 6.25 | 10238 | 3168 | 28 | 5 | 13439 | 33 | 0.002456 | 0.236 | 0.238 | 0.002459 | 0.269 | 0.272 | 0.9037% |
| B5 | 1% | 12.5 | 10692 | 8058 | 60 | 59 | 18869 | 119 | 0.006307 | 0.43 | 0.433 | 0.006327 | 0.562 | 0.568 | 1.1138% |
| B6 | 1% | 12.5 | 9340 | 6998 | 52 | 36 | 16426 | 88 | 0.005357 | 0.428 | 0.431 | 0.005372 | 0.559 | 0.565 | 0.9515% |
| C5 | 1% | 25 | 5403 | 12247 | 64 | 145 | 17859 | 209 | 0.011703 | 0.694 | 0.697 | 0.011772 | 1.184 | 1.196 | 0.9846% |
| C6 | 1% | 25 | 5183 | 11175 | 60 | 159 | 16577 | 219 | 0.013211 | 0.684 | 0.687 | 0.013299 | 1.151 | 1.163 | 1.1439% |
| D5 | 1% | 50 | 1537 | 15288 | 38 | 390 | 17253 | 428 | 0.024807 | 0.909 | 0.911 | 0.02512 | 2.394 | 2.418 | 1.0388% |
| D6 | 1% | 50 | 1149 | 12779 | 32 | 298 | 14258 | 330 | 0.023145 | 0.917 | 0.919 | 0.023417 | 2.491 | 2.518 | 0.9298% |
| E5 | 10% | 3.125 | 16567 | 2411 | 231 | 36 | 19245 | 267 | 0.013874 | 0.127 | 0.139 | 0.013971 | 0.136 | 0.15 | 9.3240% |
| E6 | 10% | 3.125 | 13580 | 1972 | 217 | 32 | 15801 | 249 | 0.015758 | 0.127 | 0.141 | 0.015884 | 0.136 | 0.151 | 10.4862% |
| F5 | 10% | 6.25 | 14424 | 4388 | 443 | 149 | 19404 | 592 | 0.030509 | 0.234 | 0.257 | 0.030984 | 0.266 | 0.297 | 10.4470% |
| F6 | 10% | 6.25 | 12979 | 3981 | 387 | 116 | 17463 | 503 | 0.028804 | 0.235 | 0.257 | 0.029227 | 0.267 | 0.297 | 9.8489% |
| G5 | 10% | 12.5 | 11249 | 7819 | 660 | 486 | 20214 | 1146 | 0.056693 | 0.411 | 0.444 | 0.058364 | 0.529 | 0.586 | 9.9581% |
| G6 | 10% | 12.5 | 5386 | 3001 | 266 | 156 | 8809 | 422 | 0.047906 | 0.358 | 0.389 | 0.049091 | 0.444 | 0.492 | 9.9784% |
| H5 | 10% | 25 | 5619 | 10118 | 709 | 1242 | 17688 | 1951 | 0.110301 | 0.642 | 0.682 | 0.116872 | 1.028 | 1.147 | 10.1917% |
| H6 | 10% | 25 | 4513 | 8907 | 562 | 1171 | 15153 | 1733 | 0.114367 | 0.665 | 0.702 | 0.121452 | 1.094 | 1.211 | 10.0271% |
| A7 | 10% | 50 | 1157 | 8434 | 308 | 2196 | 12095 | 2504 | 0.207028 | 0.879 | 0.904 | 0.231967 | 2.111 | 2.347 | 9.8837% |
| B7 | 10% | 50 | 1443 | 11598 | 367 | 3059 | 16467 | 3426 | 0.208052 | 0.89 | 0.912 | 0.23326 | 2.208 | 2.435 | 9.5809% |

***EGFR* exon 19 deletion assay**

DN - negative for VIC and FAM
DP - positive for VIC and FAM
MUT - positive for VIC only

| Well | VAF | Conc | DN | DP | MUT | TOTAL | P(MUT) | P(WT) | P(ALL) | λ(MUT) | λ(WT) | λ(ALL) | VAF(Pois) |
| --- | --- | --- | --- | --- | --- | --- | --- | --- | --- | --- | --- | --- | --- |
| A1 | 0% | 3.125 | 13036 | 1962 | 0 | 14998 | 0 | 0.131 | 0.131 | 0 | 0.14 | 0.14 | 0.000% |
| A2 | 0% | 3.125 | 15356 | 2215 | 0 | 17571 | 0 | 0.126 | 0.126 | 0 | 0.135 | 0.135 | 0.000% |
| B1 | 0% | 6.25 | 12818 | 4234 | 0 | 17052 | 0 | 0.248 | 0.248 | 0 | 0.285 | 0.285 | 0.000% |
| B2 | 0% | 6.25 | 14259 | 4504 | 0 | 18763 | 0 | 0.24 | 0.24 | 0 | 0.274 | 0.274 | 0.000% |
| C1 | 0% | 12.5 | 8210 | 6293 | 0 | 14503 | 0 | 0.434 | 0.434 | 0 | 0.569 | 0.569 | 0.000% |
| C2 | 0% | 12.5 | 9494 | 6757 | 0 | 16251 | 0 | 0.416 | 0.416 | 0 | 0.537 | 0.537 | 0.000% |
| D1 | 0% | 25 | 4772 | 10747 | 0 | 15519 | 0 | 0.693 | 0.693 | 0 | 1.179 | 1.179 | 0.000% |
| D2 | 0% | 25 | 4952 | 10723 | 0 | 15675 | 0 | 0.684 | 0.684 | 0 | 1.152 | 1.152 | 0.000% |
| E1 | 0% | 50 | 1370 | 12268 | 0 | 13638 | 0 | 0.9 | 0.9 | 0 | 2.298 | 2.298 | 0.000% |
| E2 | 0% | 50 | 1482 | 13924 | 0 | 15406 | 0 | 0.904 | 0.904 | 0 | 2.341 | 2.341 | 0.000% |
| F1 | 0.01% | 3.125 | 13723 | 2019 | 0 | 15742 | 0 | 0.128 | 0.128 | 0 | 0.137 | 0.137 | 0.000% |
| F2 | 0.01% | 3.125 | 13370 | 1971 | 0 | 15341 | 0 | 0.128 | 0.128 | 0 | 0.138 | 0.138 | 0.000% |
| G1 | 0.01% | 6.25 | 12843 | 3825 | 0 | 16668 | 0 | 0.229 | 0.229 | 0 | 0.261 | 0.261 | 0.000% |
| G2 | 0.01% | 6.25 | 13170 | 4509 | 0 | 17679 | 0 | 0.255 | 0.255 | 0 | 0.294 | 0.294 | 0.000% |
| H1 | 0.01% | 12.5 | 7756 | 5795 | 1 | 13552 | 0.00013 | 0.428 | 0.428 | 0.00013 | 0.558 | 0.558 | 0.023% |
| H2 | 0.01% | 12.5 | 9990 | 6812 | 1 | 16803 | 0.0001 | 0.405 | 0.405 | 0.0001 | 0.52 | 0.52 | 0.019% |
| A3 | 0.01% | 25 | 5303 | 10904 | 1 | 16208 | 0.00019 | 0.673 | 0.673 | 0.00019 | 1.117 | 1.117 | 0.017% |
| A4 | 0.01% | 25 | 5835 | 12038 | 0 | 17873 | 0 | 0.674 | 0.674 | 0 | 1.119 | 1.119 | 0.000% |
| B3 | 0.01% | 50 | 1747 | 14423 | 0 | 16170 | 0 | 0.892 | 0.892 | 0 | 2.225 | 2.225 | 0.000% |
| B4 | 0.01% | 50 | 1858 | 15078 | 0 | 16936 | 0 | 0.89 | 0.89 | 0 | 2.21 | 2.21 | 0.000% |
| C3 | 0.1% | 3.125 | 15310 | 2211 | 2 | 17523 | 0.00013 | 0.126 | 0.126 | 0.00013 | 0.135 | 0.135 | 0.097% |
| C4 | 0.1% | 3.125 | 13527 | 1993 | 2 | 15522 | 0.00015 | 0.128 | 0.129 | 0.00015 | 0.137 | 0.138 | 0.107% |
| D3 | 0.1% | 6.25 | 13453 | 4442 | 1 | 17896 | 7.4E-05 | 0.248 | 0.248 | 7.4E-05 | 0.285 | 0.285 | 0.026% |
| D4 | 0.1% | 6.25 | 14820 | 4861 | 9 | 19690 | 0.00061 | 0.247 | 0.247 | 0.00061 | 0.284 | 0.284 | 0.214% |
| E3 | 0.1% | 12.5 | 9916 | 7319 | 10 | 17245 | 0.00101 | 0.424 | 0.425 | 0.00101 | 0.552 | 0.553 | 0.182% |
| E4 | 0.1% | 12.5 | 9584 | 7882 | 5 | 17471 | 0.00052 | 0.451 | 0.451 | 0.00052 | 0.6 | 0.6 | 0.087% |
| F3 | 0.1% | 25 | 4840 | 11419 | 5 | 16264 | 0.00103 | 0.702 | 0.702 | 0.00103 | 1.211 | 1.212 | 0.085% |
| F4 | 0.1% | 25 | 6143 | 13299 | 13 | 19455 | 0.00211 | 0.684 | 0.684 | 0.00211 | 1.151 | 1.153 | 0.183% |
| G3 | 0.1% | 50 | 1765 | 15992 | 4 | 17761 | 0.00226 | 0.9 | 0.901 | 0.00226 | 2.307 | 2.309 | 0.098% |
| G4 | 0.1% | 50 | 1748 | 15060 | 9 | 16817 | 0.00512 | 0.896 | 0.896 | 0.00514 | 2.259 | 2.264 | 0.227% |
| H3 | 1% | 3.125 | 16490 | 2625 | 28 | 19143 | 0.0017 | 0.137 | 0.139 | 0.0017 | 0.147 | 0.149 | 1.137% |
| H4 | 1% | 3.125 | 16619 | 2507 | 21 | 19147 | 0.00126 | 0.131 | 0.132 | 0.00126 | 0.14 | 0.142 | 0.892% |
| A5 | 1% | 6.25 | 12718 | 4676 | 36 | 17430 | 0.00282 | 0.268 | 0.27 | 0.00283 | 0.312 | 0.315 | 0.897% |
| A6 | 1% | 6.25 | 13808 | 4390 | 51 | 18249 | 0.00368 | 0.241 | 0.243 | 0.00369 | 0.275 | 0.279 | 1.322% |
| B5 | 1% | 12.5 | 8150 | 6232 | 42 | 14424 | 0.00513 | 0.432 | 0.435 | 0.00514 | 0.566 | 0.571 | 0.900% |
| B6 | 1% | 12.5 | 10444 | 7872 | 65 | 18381 | 0.00619 | 0.428 | 0.432 | 0.0062 | 0.559 | 0.565 | 1.098% |
| C5 | 1% | 25 | 5706 | 12324 | 72 | 18102 | 0.01246 | 0.681 | 0.685 | 0.01254 | 1.142 | 1.155 | 1.086% |
| C6 | 1% | 25 | 5951 | 12234 | 70 | 18255 | 0.01163 | 0.67 | 0.674 | 0.01169 | 1.109 | 1.121 | 1.043% |
| D5 | 1% | 50 | 1564 | 15218 | 43 | 16825 | 0.02676 | 0.904 | 0.907 | 0.02712 | 2.348 | 2.376 | 1.142% |
| D6 | 1% | 50 | 1762 | 15976 | 40 | 17778 | 0.0222 | 0.899 | 0.901 | 0.02245 | 2.289 | 2.312 | 0.971% |
| E5 | 10% | 3.125 | 14236 | 1978 | 198 | 16412 | 0.01372 | 0.121 | 0.133 | 0.01381 | 0.128 | 0.142 | 9.711% |
| E6 | 10% | 3.125 | 17520 | 2429 | 245 | 20194 | 0.01379 | 0.12 | 0.132 | 0.01389 | 0.128 | 0.142 | 9.777% |
| F5 | 10% | 6.25 | 11844 | 3629 | 359 | 15832 | 0.02942 | 0.229 | 0.252 | 0.02986 | 0.26 | 0.29 | 10.289% |
| F6 | 10% | 6.25 | 13366 | 4177 | 412 | 17955 | 0.0299 | 0.233 | 0.256 | 0.03036 | 0.265 | 0.295 | 10.286% |
| G5 | 10% | 12.5 | 11124 | 7875 | 630 | 19629 | 0.0536 | 0.401 | 0.433 | 0.05509 | 0.513 | 0.568 | 9.700% |
| G6 | 10% | 12.5 | 9186 | 6521 | 553 | 16260 | 0.05678 | 0.401 | 0.435 | 0.05846 | 0.513 | 0.571 | 10.237% |
| H5 | 10% | 25 | 4744 | 10890 | 627 | 16261 | 0.11674 | 0.67 | 0.708 | 0.12413 | 1.108 | 1.232 | 10.077% |
| H6 | 10% | 25 | 5385 | 10772 | 643 | 16800 | 0.10667 | 0.641 | 0.679 | 0.1128 | 1.025 | 1.138 | 9.914% |
| A7 | 10% | 50 | 1512 | 13149 | 424 | 15085 | 0.21901 | 0.872 | 0.9 | 0.24719 | 2.053 | 2.3 | 10.746% |
| B7 | 10% | 50 | 1307 | 13847 | 434 | 15588 | 0.24928 | 0.888 | 0.916 | 0.28673 | 2.192 | 2.479 | 11.567% |

**Turnaround Time**

The data used to calculate turnaround time statistics (Fig 6).

| Test ID | Test Type | Turnaround Time (Days) |
| --- | --- | --- |
| 1 | ddPCR | 3 |
| 2 | ddPCR | 10 |
| 3 | ddPCR | 4 |
| 4 | ddPCR | 4 |
| 5 | ddPCR | 4 |
| 6 | ddPCR | 3 |
| 7 | ddPCR | 8 |
| 8 | ddPCR | 8 |
| 9 | ddPCR | 8 |
| 10 | ddPCR | 7 |
| 11 | ddPCR | 7 |
| 12 | ddPCR | 8 |
| 13 | ddPCR | 7 |
| 14 | ddPCR | 5 |
| 15 | ddPCR | 4 |
| 16 | ddPCR | 7 |
| 17 | ddPCR | 1 |
| 18 | ddPCR | 5 |
| 19 | ddPCR | 3 |
| 20 | ddPCR | 3 |
| 21 | ddPCR | 4 |
| 22 | ddPCR | 5 |
| 23 | ddPCR | 5 |
| 24 | ddPCR | 3 |
| 25 | ddPCR | 5 |
| 26 | ddPCR | 5 |
| 27 | ddPCR | 5 |
| 28 | ddPCR | 6 |
| 29 | ddPCR | 6 |
| 30 | ddPCR | 6 |
| 31 | ddPCR | 6 |
| 32 | ddPCR | 6 |
| 33 | ddPCR | 3 |
| 34 | ddPCR | 3 |
| 35 | ddPCR | 4 |
| 36 | ddPCR | 6 |
| 37 | ddPCR | 6 |
| 38 | ddPCR | 6 |
| 39 | ddPCR | 6 |
| 40 | ddPCR | 5 |
| 41 | ddPCR | 3 |
| 42 | ddPCR | 3 |
| 43 | ddPCR | 1 |
| 44 | ddPCR | 6 |
| 45 | ddPCR | 5 |
| 46 | ddPCR | 4 |
| 47 | ddPCR | 9 |
| 48 | ddPCR | 2 |
| 49 | ddPCR | 4 |
| 50 | ddPCR | 2 |
| 51 | ddPCR | 4 |
| 52 | ddPCR | 3 |
| 53 | ddPCR | 5 |
| 54 | ddPCR | 7 |
| 55 | ddPCR | 5 |
| 56 | ddPCR | 1 |
| 57 | ddPCR | 7 |
| 58 | ddPCR | 2 |
| 59 | ddPCR | 6 |
| 60 | ddPCR | 3 |
| 61 | ddPCR | 3 |
| 62 | ddPCR | 7 |
| 63 | ddPCR | 1 |
| 64 | ddPCR | 6 |
| 65 | ddPCR | 2 |
| 66 | ddPCR | 3 |
| 67 | ddPCR | 3 |
| 68 | ddPCR | 3 |
| 69 | ddPCR | 5 |
| 70 | ddPCR | 8 |
| 71 | ddPCR | 2 |
| 72 | ddPCR | 2 |
| 73 | ddPCR | 3 |
| 74 | ddPCR | 2 |
| 75 | ddPCR | 1 |
| 76 | ddPCR | 6 |
| 77 | ddPCR | 4 |
| 78 | ddPCR | 4 |
| 79 | ddPCR | 5 |
| 80 | ddPCR | 6 |
| 81 | ddPCR | 3 |
| 82 | ddPCR | 5 |
| 83 | ddPCR | 4 |
| 84 | ddPCR | 5 |
| 85 | ddPCR | 4 |
| 86 | ddPCR | 8 |
| 87 | ddPCR | 6 |
| 88 | ddPCR | 2 |
| 89 | ddPCR | 3 |
| 90 | ddPCR | 3 |
| 91 | ddPCR | 7 |
| 92 | ddPCR | 1 |
| 93 | ddPCR | 2 |
| 94 | ddPCR | 2 |
| 95 | ddPCR | 6 |
| 96 | ddPCR | 2 |
| 97 | ddPCR | 4 |
| 98 | ddPCR | 5 |
| 99 | ddPCR | 9 |
| 100 | ddPCR | 5 |
| 101 | ddPCR | 9 |
| 102 | ddPCR | 6 |
| 103 | ddPCR | 6 |
| 104 | ddPCR | 1 |
| 105 | ddPCR | 5 |
| 106 | ddPCR | 1 |
| 107 | ddPCR | 6 |
| 108 | ddPCR | 5 |
| 109 | ddPCR | 5 |
| 110 | ddPCR | 6 |
| 111 | ddPCR | 6 |
| 112 | ddPCR | 7 |
| 113 | ddPCR | 4 |
| 114 | ddPCR | 7 |
| 115 | ddPCR | 2 |
| 116 | ddPCR | 5 |
| 117 | ddPCR | 4 |
| 118 | ddPCR | 6 |
| 119 | ddPCR | 5 |
| 120 | ddPCR | 5 |
| 121 | ddPCR | 3 |
| 122 | ddPCR | 6 |
| 123 | ddPCR | 3 |
| 124 | ddPCR | 2 |
| 125 | ddPCR | 9 |
| 126 | ddPCR | 1 |
| 127 | ddPCR | 8 |
| 128 | ddPCR | 4 |
| 129 | ddPCR | 11 |
| 130 | ddPCR | 2 |
| 131 | ddPCR | 2 |
| 132 | ddPCR | 2 |
| 133 | ddPCR | 2 |
| 134 | ddPCR | 4 |
| 135 | ddPCR | 11 |
| 136 | ddPCR | 5 |
| 137 | ddPCR | 6 |
| 138 | ddPCR | 4 |
| 139 | ddPCR | 4 |
| 140 | ddPCR | 2 |
| 141 | ddPCR | 3 |
| 142 | ddPCR | 3 |
| 143 | ddPCR | 7 |
| 144 | ddPCR | 9 |
| 145 | ddPCR | 4 |
| 146 | ddPCR | 5 |
| 147 | ddPCR | 5 |
| 148 | ddPCR | 4 |
| 149 | ddPCR | 4 |
| 150 | ddPCR | 6 |
| 151 | ddPCR | 3 |
| 152 | ddPCR | 7 |
| 153 | ddPCR | 4 |
| 154 | ddPCR | 3 |
| 155 | ddPCR | 2 |
| 156 | ddPCR | 6 |
| 157 | ddPCR | 5 |
| 158 | ddPCR | 1 |
| 159 | ddPCR | 5 |
| 160 | ddPCR | 4 |
| 161 | ddPCR | 4 |
| 162 | ddPCR | 4 |
| 163 | ddPCR | 9 |
| 164 | ddPCR | 3 |
| 165 | ddPCR | 3 |
| 166 | ddPCR | 4 |
| 167 | ddPCR | 2 |
| 168 | ddPCR | 3 |
| 169 | ddPCR | 11 |
| 170 | ddPCR | 5 |
| 171 | ddPCR | 4 |
| 172 | ddPCR | 8 |
| 173 | ddPCR | 3 |
| 174 | ddPCR | 6 |
| 175 | ddPCR | 1 |
| 176 | ddPCR | 4 |
| 177 | ddPCR | 7 |
| 178 | ddPCR | 4 |
| 179 | ddPCR | 3 |
| 180 | ddPCR | 4 |
| 181 | ddPCR | 9 |
| 182 | ddPCR | 5 |
| 183 | ddPCR | 10 |
| 184 | ddPCR | 7 |
| 185 | ddPCR | 8 |
| 186 | ddPCR | 5 |
| 187 | ddPCR | 5 |
| 188 | ddPCR | 4 |
| 189 | ddPCR | 3 |
| 190 | ddPCR | 8 |
| 191 | ddPCR | 2 |
| 192 | ddPCR | 4 |
| 193 | ddPCR | 6 |
| 194 | ddPCR | 8 |
| 195 | ddPCR | 3 |
| 196 | ddPCR | 4 |
| 197 | ddPCR | 5 |
| 198 | ddPCR | 2 |
| 199 | ddPCR | 5 |
| 200 | ddPCR | 9 |
| 201 | ddPCR | 1 |
| 202 | ddPCR | 2 |
| 203 | ddPCR | 7 |
| 204 | ddPCR | 5 |
| 205 | ddPCR | 2 |
| 206 | ddPCR | 9 |
| 207 | ddPCR | 5 |
| 208 | ddPCR | 5 |
| 209 | ddPCR | 4 |
| 210 | ddPCR | 6 |
| 211 | ddPCR | 5 |
| 212 | ddPCR | 4 |
| 213 | ddPCR | 7 |
| 214 | ddPCR | 6 |
| 215 | ddPCR | 6 |
| 216 | ddPCR | 6 |
| 217 | ddPCR | 7 |
| 218 | ddPCR | 4 |
| 219 | ddPCR | 3 |
| 220 | ddPCR | 3 |
| 221 | ddPCR | 2 |
| 222 | ddPCR | 4 |
| 223 | ddPCR | 4 |
| 224 | ddPCR | 3 |
| 225 | ddPCR | 6 |
| 226 | ddPCR | 4 |
| 227 | ddPCR | 7 |
| 228 | ddPCR | 2 |
| 229 | ddPCR | 1 |
| 230 | ddPCR | 6 |
| 231 | ddPCR | 5 |
| 232 | ddPCR | 7 |
| 233 | ddPCR | 6 |
| 234 | ddPCR | 4 |
| 235 | ddPCR | 1 |
| 236 | ddPCR | 5 |
| 237 | ddPCR | 6 |
| 238 | ddPCR | 9 |
| 239 | ddPCR | 4 |
| 240 | ddPCR | 10 |
| 241 | ddPCR | 2 |
| 242 | ddPCR | 7 |
| 243 | ddPCR | 6 |
| 244 | ddPCR | 4 |
| 245 | ddPCR | 6 |
| 246 | ddPCR | 5 |
| 247 | ddPCR | 6 |
| 248 | ddPCR | 6 |
| 249 | ddPCR | 6 |
| 250 | ddPCR | 1 |
| 251 | ddPCR | 6 |
| 252 | ddPCR | 3 |
| 253 | ddPCR | 5 |
| 254 | ddPCR | 4 |
| 255 | NGS | 15 |
| 256 | NGS | 14 |
| 257 | NGS | 11 |
| 258 | NGS | 12 |
| 259 | NGS | 20 |
| 260 | NGS | 28 |
| 261 | NGS | 14 |
| 262 | NGS | 14 |
| 263 | NGS | 19 |
| 264 | NGS | 12 |
| 265 | NGS | 11 |
| 266 | NGS | 9 |
| 267 | NGS | 13 |
| 268 | NGS | 13 |
| 269 | NGS | 8 |
| 270 | NGS | 12 |
| 271 | NGS | 13 |
| 272 | NGS | 9 |
| 273 | NGS | 14 |
| 274 | NGS | 11 |
| 275 | NGS | 32 |
| 276 | NGS | 13 |
| 277 | NGS | 16 |
| 278 | NGS | 9 |
| 279 | NGS | 9 |
| 280 | NGS | 14 |
| 281 | NGS | 13 |
| 282 | NGS | 13 |
| 283 | NGS | 19 |
| 284 | NGS | 13 |
| 285 | NGS | 8 |
| 286 | NGS | 8 |
| 287 | NGS | 13 |
| 288 | NGS | 11 |
| 289 | NGS | 12 |
| 290 | NGS | 16 |
| 291 | NGS | 12 |
| 292 | NGS | 13 |
| 293 | NGS | 13 |
| 294 | NGS | 13 |
| 295 | NGS | 13 |
| 296 | NGS | 14 |
| 297 | NGS | 11 |
| 298 | NGS | 9 |
| 299 | NGS | 13 |
| 300 | NGS | 11 |
| 301 | NGS | 20 |
| 302 | NGS | 7 |
| 303 | NGS | 15 |
| 304 | NGS | 12 |
| 305 | NGS | 8 |
| 306 | NGS | 8 |
| 307 | NGS | 14 |
| 308 | NGS | 14 |
| 309 | NGS | 10 |
| 310 | NGS | 20 |
| 311 | NGS | 14 |
| 312 | NGS | 8 |
| 313 | NGS | 13 |
| 314 | NGS | 11 |
| 315 | NGS | 8 |
| 316 | NGS | 12 |
| 317 | NGS | 6 |
| 318 | NGS | 11 |
| 319 | NGS | 11 |
| 320 | NGS | 8 |
| 321 | NGS | 14 |
| 322 | NGS | 12 |
| 323 | NGS | 9 |
| 324 | NGS | 16 |
| 325 | NGS | 10 |
| 326 | NGS | 12 |
| 327 | NGS | 13 |
| 328 | NGS | 21 |
| 329 | NGS | 14 |
| 330 | NGS | 18 |
| 331 | NGS | 14 |
| 332 | NGS | 13 |
| 333 | NGS | 11 |
| 334 | NGS | 11 |
| 335 | NGS | 15 |
| 336 | NGS | 15 |
| 337 | NGS | 13 |
| 338 | NGS | 14 |
| 339 | NGS | 12 |
| 340 | NGS | 11 |
| 341 | NGS | 14 |
| 342 | NGS | 21 |
| 343 | NGS | 14 |
| 344 | NGS | 9 |
| 345 | NGS | 8 |
| 346 | NGS | 10 |
| 347 | NGS | 15 |
| 348 | NGS | 9 |
| 349 | NGS | 17 |
| 350 | NGS | 14 |
| 351 | NGS | 14 |
| 352 | NGS | 23 |
| 353 | NGS | 18 |
| 354 | NGS | 12 |
| 355 | NGS | 9 |
| 356 | NGS | 21 |
| 357 | NGS | 8 |
| 358 | NGS | 15 |
| 359 | NGS | 12 |
| 360 | NGS | 13 |
| 361 | NGS | 11 |
| 362 | NGS | 36 |
| 363 | NGS | 13 |
| 364 | NGS | 8 |
| 365 | NGS | 10 |
| 366 | NGS | 8 |
| 367 | NGS | 13 |
| 368 | NGS | 15 |
| 369 | NGS | 9 |
| 370 | NGS | 8 |
| 371 | NGS | 11 |
| 372 | NGS | 14 |
| 373 | NGS | 12 |
| 374 | NGS | 14 |
| 375 | NGS | 8 |
| 376 | NGS | 13 |
| 377 | NGS | 14 |
| 378 | NGS | 17 |
| 379 | NGS | 7 |
| 380 | NGS | 12 |
| 381 | NGS | 13 |
| 382 | NGS | 15 |
| 383 | NGS | 15 |
| 384 | NGS | 16 |
| 385 | NGS | 12 |
| 386 | NGS | 17 |
| 387 | NGS | 17 |
| 388 | NGS | 52 |
| 389 | NGS | 10 |
| 390 | NGS | 13 |
| 391 | NGS | 9 |
| 392 | NGS | 40 |
| 393 | NGS | 23 |
| 394 | NGS | 9 |
| 395 | NGS | 36 |
| 396 | NGS | 28 |
| 397 | NGS | 25 |
| 398 | NGS | 17 |
| 399 | NGS | 38 |
| 400 | NGS | 8 |
| 401 | NGS | 30 |
| 402 | NGS | 10 |
| 403 | NGS | 13 |
| 404 | NGS | 42 |
| 405 | NGS | 17 |
| 406 | NGS | 45 |
| 407 | NGS | 51 |
| 408 | NGS | 11 |
| 409 | NGS | 28 |
| 410 | NGS | 20 |
| 411 | NGS | 13 |
| 412 | NGS | 13 |
| 413 | NGS | 7 |
| 414 | NGS | 133 |
| 415 | NGS | 36 |
| 416 | NGS | 48 |
| 417 | NGS | 11 |
| 418 | NGS | 11 |
| 419 | NGS | 33 |
| 420 | NGS | 45 |
| 421 | NGS | 48 |
| 422 | NGS | 38 |
| 423 | NGS | 25 |
| 424 | NGS | 24 |
| 425 | NGS | 22 |
| 426 | NGS | 17 |
| 427 | NGS | 49 |
| 428 | NGS | 11 |
| 429 | NGS | 28 |
| 430 | NGS | 18 |
| 431 | NGS | 36 |
| 432 | NGS | 51 |
| 433 | NGS | 21 |
| 434 | NGS | 37 |
| 435 | NGS | 54 |
| 436 | NGS | 22 |
| 437 | NGS | 9 |
| 438 | NGS | 24 |
| 439 | NGS | 14 |
| 440 | NGS | 31 |
| 441 | NGS | 13 |
| 442 | NGS | 12 |
| 443 | NGS | 51 |
| 444 | NGS | 49 |
| 445 | NGS | 24 |
| 446 | NGS | 14 |
| 447 | NGS | 11 |
| 448 | NGS | 14 |
| 449 | NGS | 39 |
| 450 | NGS | 56 |
| 451 | NGS | 14 |
| 452 | NGS | 13 |
| 453 | NGS | 14 |
| 454 | NGS | 23 |
| 455 | NGS | 9 |
| 456 | NGS | 15 |
| 457 | NGS | 42 |
| 458 | NGS | 12 |
| 459 | NGS | 12 |
| 460 | NGS | 15 |
| 461 | NGS | 11 |
| 462 | NGS | 16 |
| 463 | NGS | 11 |
| 464 | NGS | 12 |
| 465 | NGS | 12 |
| 466 | NGS | 21 |
| 467 | NGS | 8 |
| 468 | NGS | 13 |
| 469 | NGS | 14 |
| 470 | NGS | 9 |
| 471 | NGS | 12 |
| 472 | NGS | 14 |
| 473 | NGS | 21 |
| 474 | NGS | 9 |
| 475 | NGS | 14 |
| 476 | NGS | 7 |
| 477 | NGS | 19 |
| 478 | NGS | 14 |
| 479 | NGS | 17 |
| 480 | NGS | 14 |
| 481 | NGS | 14 |
| 482 | NGS | 15 |
| 483 | NGS | 15 |
| 484 | NGS | 11 |
| 485 | NGS | 13 |
| 486 | NGS | 16 |
| 487 | NGS | 13 |
| 488 | NGS | 14 |
| 489 | NGS | 42 |
| 490 | NGS | 15 |
| 491 | NGS | 15 |
| 492 | NGS | 11 |
| 493 | NGS | 17 |
| 494 | NGS | 14 |
| 495 | NGS | 10 |
| 496 | NGS | 13 |
| 497 | NGS | 16 |
| 498 | NGS | 12 |
| 499 | NGS | 22 |
| 500 | NGS | 11 |
| 501 | NGS | 12 |
| 502 | NGS | 11 |
| 503 | NGS | 10 |
| 504 | NGS | 16 |
| 505 | NGS | 18 |
| 506 | NGS | 14 |
| 507 | NGS | 21 |
| 508 | NGS | 14 |
| 509 | NGS | 14 |
| 510 | NGS | 8 |
| 511 | NGS | 13 |
| 512 | NGS | 28 |
| 513 | NGS | 12 |
| 514 | NGS | 7 |
| 515 | NGS | 12 |
| 516 | NGS | 12 |
| 517 | NGS | 11 |
| 518 | NGS | 8 |
| 519 | NGS | 10 |
| 520 | NGS | 16 |

**Plasma ddPCR vs NGS concordance**

The data used to calculate sensitivity of the ddPCR assay performed on plasma as well as associated clinical characteristics for each patient. Clinical characteristics determined at the time of ddPCR test.

TAT – Turnaround time

Ex19del – exon 19 deletion

Tx - Treatment

| Patient | ddPCR TAT | ddPCR L858R result | ddPCR ex19del result | NGS TAT | NGS L858R result | NGS ex19del result | Age | Sex | Stage | Status (New/Recur) | Metastasis | Site | Treatment | Tx Type |
| --- | --- | --- | --- | --- | --- | --- | --- | --- | --- | --- | --- | --- | --- | --- |
| 1 | 3 | Pos | Neg | 14 | Pos | Neg | 64 | F | 4 | New | Y | Lymph | N | None |
| 2 | 6 | Neg | Neg | 13 | Pos | Neg | 72 | F | 4 | Recur | Y | Lung | Y | Surgery |
| 3 | 2 | Pos | Neg | 7 | Pos | Neg | 64 | F | 4 | New | Y | Lymph, Brain, Bone | N | None |
| 4 | 1 | Pos | Neg | 21 | Pos | Neg | 55 | M | 4 | New | Y | Brain, Bone | N | None |
| 5 | 6 | Neg | Neg | 13 | Pos | Neg | 79 | F | 4 | Recur | N | None | Y | Surgery, TKI |
| 6 | 5 | Pos | Neg | 14 | Pos | Neg | 48 | M | 4 | New | Y | Lymph, Brain, Bone, Liver | N | None |
| 7 | 1 | Pos | Neg | 10 | Pos | Neg | 60 | M | 4 | New | Y | Lymph, Brain, Bone, Liver | N | None |
| 8 | 6 | Pos | Neg | 21 | Pos | Neg | 87 | F | 4 | New | Y | Lymph, Bone, Soft tissue | N | None |
| 9 | 6 | Pos | Neg | 19 | Pos | Neg | 60 | F | 4 | Recur | Y | Lymph | Y | TKI |
| 10 | 11 | Pos | Neg | 14 | Pos | Neg | 47 | F | 4 | Recur | Y | Liver, Bone | Y | Chemo |
| 11 | 2 | Pos | Neg | 12 | Pos | Neg | 53 | M | 4 | New | Y | Lymph, Bone, Brain | N | None |
| 12 | 2 | Pos | Neg | 17 | Pos | Neg | 58 | F | 4 | Recur | Y | Lymph | Y | Rads |
| 13 | 4 | Pos | Neg | 52 | Pos | Neg | 65 | F | 4 | Recur | Y | Lymph, Bone, Brain, Lung | Y | Surgery, Chemo, TKI |
| 14 | 11 | Neg | Neg | 21 | Pos | Neg | 56 | F | 4 | Recur | Y | Brain, Bone | Y | TKI |
| 15 | 6 | Pos | Neg | 14 | Pos | Neg | 70 | M | 4 | Recur | Y | Lymph | Y | TKI |
| 16 | 9 | Neg | Neg | 17 | Pos | Neg | 55 | M | 4 | Recur | Y | Lymph, Bone | Y | Surgery, TKI |
| 17 | 4 | Neg | Neg | 52 | Pos | Neg | 41 | F | 4 | Recur | Y | Lymph, Bone, Soft tissue | Y | IO |
| 18 | 3 | Neg | Neg | 11 | Pos | Neg | 81 | M | 4 | Recur | Y | Bone | Y | TKI |
| 19 | 2 | Neg | Neg | 28 | Pos | Neg | 56 | F | 4 | Recur | Y | Brain, Lung | Y | TKI |
| 20 | 5 | Pos | Neg | 7 | Pos | Neg | 75 | F | 4 | Recur | Y | Lung, Liver, Bone | Y | TKI |
| 21 | 4 | Pos | Neg | 36 | Pos | Neg | 65 | M | 4 | Recur | Y | Lymph, Bone, Brain | Y | Chemo, TKI |
| 22 | 9 | Neg | Neg | 11 | Pos | Neg | 62 | M | 4 | Recur | Y | Lymph | Y | TKI |
| 23 | 11 | Pos | Neg | 14 | Pos | Neg | 73 | F | 4 | Recur | Y | Lymph, Bone, Soft tissue | Y | TKI |
| 24 | 3 | Neg | Neg | 49 | Pos | Neg | 78 | F | 4 | Recur | Y | Pleura | Y | Rads, TKI |
| 25 | 1 | Neg | Neg | 28 | Pos | Neg | 69 | F | 4 | Recur | Y | Pleura | Y | Surgery, TKI |
| 26 | 4 | Pos | Neg | 19 | Pos | Neg | 82 | M | 3C | Recur | Y | Lymph | Y | Chemo, Rads |
| 27 | 4 | Pos | Neg | 51 | Pos | Neg | 95 | F | 4 | Recur | Y | Pleura | Y | Rads, TKI |
| 28 | 3 | Pos | Neg | 21 | Pos | Neg | 65 | F | 4 | Recur | Y | Lymph, Bone | Y | IO, Chemo, TKI |
| 29 | 5 | Neg | Neg | 22 | Pos | Neg | 68 | M | 4 | Recur | Y | Brain | Y | Surgery, Chemo, Rads, TKI |
| 30 | 8 | Pos | Neg | 14 | Pos | Neg | 53 | F | 4 | Recur | Y | Bone | Y | TKI |
| 31 | 4 | Pos | Neg | 12 | Pos | Neg | 64 | F | 4 | Recur | Y | Bone | Y | TKI |
| 32 | 3 | Neg | Neg | 11 | Pos | Neg | 78 | F | 4 | Recur | Y | Pleura | Y | TKI |
| 33 | 8 | Neg | Neg | 14 | Pos | Neg | 59 | F | 4 | Recur | Y | Pleural effusion | Y | Surgery, TKI |
| 34 | 4 | Pos | Neg | 56 | Pos | Neg | 60 | N | 4 | Recur | Y | Bone | Y | Chemo, IO, TKI |
| 35 | 2 | Neg | Neg | 39 | Pos | Neg | 77 | M | 4 | Recur | Y | Bone | Y | Surgery, Chemo, TKI |
| 36 | 2 | Pos | Neg | 12 | Pos | Neg | 74 | F | 4 | New | Y | Lymph, Bone | N | None |
| 37 | 3 | Neg | Neg | 15 | Neg | Pos | 56 | F | 4 | New | Y | Lymph, Bone | N | None |
| 38 | 4 | Neg | Pos | 20 | Neg | Pos | 84 | F | 4 | New | Y | Bone, Brain | N | None |
| 39 | 6 | Neg | Neg | 19 | Neg | Pos | 51 | M | 3 | New | N | None | N | None |
| 40 | 6 | Neg | Pos | 8 | Neg | Pos | 70 | M | 4 | Recur | Y | Lymph | Y | Surgery, Chemo |
| 41 | 5 | Neg | Pos | 13 | Neg | Pos | 61 | F | 4 | New | Y | Pleura | N | None |
| 42 | 2 | Neg | Neg | 12 | Neg | Pos | 62 | F | 4 | Recur | Y | Kidney | Y | Surgery, Chemo |
| 43 | 4 | Neg | Pos | 8 | Neg | Pos | 81 | F | 4 | New | Y | Bone | N | None |
| 44 | 5 | Neg | Neg | 10 | Neg | Pos | 47 | M | 4 | New | Y | Pleural effusion, Brain | N | None |
| 45 | 7 | Neg | Neg | 12 | Neg | Pos | 51 | M | 4 | New | Y | Pleural effusion | N | None |
| 46 | 1 | Neg | Pos | 7 | Neg | Pos | 61 | F | 4 | New | Y | Bone | N | None |
| 47 | 3 | Neg | Pos | 12 | Neg | Pos | 71 | M | 4 | New | Y | Bone | N | None |
| 48 | 8 | Neg | Neg | 16 | Neg | Pos | 87 | M | 4 | Recur | Y | Bone | Y | TKI |
| 49 | 2 | Neg | Pos | 21 | Neg | Pos | 64 | M | 4 | Recur | Y | Bone, Soft tissue | Y | Chemo, Rads, TKI |
| 50 | 1 | Neg | Pos | 14 | Neg | Pos | 61 | M | 4 | New | Y | Bone, Liver, Adrenal, Omentum, Lymph | N | None |
| 51 | 4 | Neg | Pos | 13 | Neg | Pos | 49 | F | 4 | New | Y | Lymph, Liver, Pleural effusion | N | None |
| 52 | 3 | Neg | Pos | 15 | Neg | Pos | 31 | F | 4 | Recur | Y | Liver, Bone | Y | Rads, TKI |
| 53 | 8 | Neg | Pos | 12 | Neg | Pos | 47 | F | 4 | Recur | Y | Liver, Bone, Brain, Pleural effusion | Y | Surgery, Rads, TKI |
| 54 | 6 | Neg | Pos | 14 | Neg | Pos | 61 | F | 4 | New | Y | Liver, Bone, Brain, Pleura | N | None |
| 55 | 3 | Neg | Pos | 14 | Neg | Pos | 69 | F | 4 | Recur | Y | Lymph, Bone | Y | Chemo, Rads |
| 56 | 6 | Neg | Pos | 17 | Neg | Pos | 80 | F | 4 | New | Y | Lymph, Liver, Bone, Brain, Pancreas, Adrenal | N | None |
| 57 | 2 | Neg | Neg | 14 | Neg | Pos | 51 | F | 3A | Recur | N | None | Y | Chemo, Rads, Surgery, TKI |
| 58 | 9 | Neg | Neg | 9 | Neg | Pos | 68 | F | 4 | Recur | Y | Lung, Brain | Y | TKI |
| 59 | 5 | Neg | Neg | 12 | Neg | Pos | 58 | F | 4 | Recur | Y | Brain, Bone | Y | TKI |
| 60 | 6 | Neg | Neg | 11 | Neg | Pos | 84 | F | 4 | Recur | Y | Brain | Y | TKI |
| 61 | 4 | Neg | Pos | 13 | Neg | Pos | 52 | F | 4 | New | Y | Liver, Bone | N | None |
| 62 | 7 | Neg | Neg | 15 | Neg | Pos | 67 | F | "3-4" | New | Unknown | Unknown | N | None |
| 63 | 5 | Neg | Neg | 12 | Neg | Pos | 47 | F | 4 | Recur | Y | Lung, Lymph | Y | TKI |
| 64 | 5 | Neg | Neg | 14 | Neg | Pos | 32 | F | 4 | New | Y | Pleural effusion | N | None |
| 65 | 8 | Neg | Neg | 13 | Neg | Pos | 44 | M | 3A | New | N | None | N | None |
| 66 | 4 | Neg | Pos | 15 | Neg | Pos | 54 | F | 4 | Recur | Y | Bone, Liver, Pleural effusion | Y | Surgery |
| 67 | 2 | Neg | Pos | 17 | Neg | Pos | 66 | F | 4 | New | Y | Bone, Brain | N | None |
| 68 | 3 | Neg | Neg | 15 | Neg | Pos | 67 | F | 4 | Recur | Y | Lung, Bone | Y | TKI |
| 69 | 7 | Neg | Pos | 25 | Neg | Pos | 52 | F | 4 | New | Y | Lymph, Bone, Brain | N | None |
| 70 | 5 | Neg | Neg | 31 | Neg | Pos | 62 | F | 4 | Recur | Y | Brain, Lung, Pleural fluid | Y | Chemo, TKI |
| 71 | 4 | Neg | Neg | 10 | Neg | Pos | 71 | F | 4 | Recur | Y | Lymph, Brain | Y | TKI |
| 72 | 4 | Neg | Pos | 13 | Neg | Pos | 66 | F | 4 | Recur | Y | Bone | Y | TKI |
| 73 | 6 | Neg | Neg | 43 | Neg | Pos | 70 | M | 4 | Recur | Y | Lung, Pleural effusion | Y | TKI |
| 74 | 7 | Neg | Neg | 46 | Neg | Pos | 48 | F | 3B | Recur | Y | Lymph | Y | Surgery, TKI |
| 75 | 6 | Neg | Pos | 20 | Neg | Pos | 57 | F | 4 | Recur | Y | Bone | Y | Surgery, Chemo, TKI |
| 76 | 4 | Neg | Neg | 133 | Neg | Pos | 82 | F | 4 | Recur | Y | Lung, Liver | Y | TKI |
| 77 | 4 | Neg | Pos | 48 | Neg | Pos | 47 | F | 4 | Recur | Y | Liver, Bone | Y | Chemo, Rads, TKI |
| 78 | 3 | Neg | Neg | 11 | Neg | Pos | 77 | F | 3A | Recur | Y | Lymph | Y | Chemo, Rads |
| 79 | 2 | Neg | Pos | 17 | Neg | Pos | 58 | F | 4 | Recur | Y | Brain, Bone, Adrenal, Kidney, Lymph | Y | TKI |
| 80 | 3 | Neg | Pos | 38 | Neg | Pos | 66 | M | 4 | Recur | Y | Brain, Bone | Y | TKI |
| 81 | 4 | Neg | Pos | 22 | Neg | Pos | 66 | F | 4 | Recur | Y | Bone | Y | TKI |
| 82 | 7 | Neg | Neg | 36 | Neg | Pos | 58 | M | 4 | Recur | Y | Pleura, Lymph | Y | Surgery, Chemo, Rads, TKI |
| 83 | 4 | Neg | Pos | 37 | Neg | Pos | 58 | F | 4 | Recur | Y | Adrenal | Y | Chemo, Rads, TKI |
| 84 | 10 | Neg | Neg | 9 | Neg | Pos | 65 | F | 4 | Recur | Y | Bone, Brain | Y | TKI |
| 85 | 8 | Neg | Pos | 24 | Neg | Pos | 73 | F | 4 | Recur | Y | Lung, Bone | Y | Chemo, Rads, TKI |
| 86 | 6 | Neg | Neg | 23 | Neg | Pos | 68 | M | 4 | Recur | Y | Brain | Y | Rads, TKI |
| 87 | 8 | Neg | Neg | 49 | Neg | Pos | 71 | F | 1A | Recur | N | None | Y | Surgery |
| 88 | 2 | Neg | Neg | 24 | Neg | Pos | 97 | F | 1B | Recur | N | None | Y | Surgery, TKI |
| 89 | 4 | Neg | Neg | 14 | Neg | Pos | 74 | M | 4 | Recur | Y | Bone | Y | Surgery, TKI |
| 90 | 2 | Neg | Neg | 13 | Neg | Pos | 91 | F | 4 | New | Y | Pleural effusion | N | None |
| 91 | 9 | Neg | Pos | 23 | Neg | Pos | 57 | F | 4 | Recur | Y | Brain | Y | Chemo, TKI |
| 92 | 5 | Neg | Neg | 11 | Neg | Pos | 69 | F | 2 | Recur | N | None | Y | Surgery |
